# Photothermal effect of gold nanostar patterns inkjet-printed on coated paper substrates with different permeability

**DOI:** 10.3762/bjnano.7.140

**Published:** 2016-10-19

**Authors:** Mykola Borzenkov, Anni Määttänen, Petri Ihalainen, Maddalena Collini, Elisa Cabrini, Giacomo Dacarro, Piersandro Pallavicini, Giuseppe Chirico

**Affiliations:** 1 Department of Physics “G. Occhialini”, Nanomedicine Center, University of Milano-Bicocca, piazza dell’Ateneo Nuovo, 20126, Milan, Italy; 2 Laboratory of Physical Chemistry, Center of Functional Materials, Åbo Academi University, Porthaninkatu 3-5, 20500, Turku, Finland; 3 Department of Chemistry, University of Pavia, viale Taramelli 12, 27100, Pavia, Italy

**Keywords:** gold nanostars, inkjet printing, localized surface plasmon resonance (LSPR), photothermal effect

## Abstract

Inkjet printing of spherical gold nanoparticles is widely applied in the fabrication of analytical and diagnostics tools. These methods could be extended to non-spherical gold nanoparticles that can efficiently release heat locally when irradiated in the near infrared (NIR) wavelength region, due to localized surface plasmon resonance (LSPR). However, this promising application requires the ability to maintain high efficiency and tunability of the NIR LSPR of the printed nanoparticles. In this study stable inks containing PEGylated gold nanostars (GNS) were fabricated and successfully inkjet-printed onto differently coated paper substrates with different porosity and permeability. A pronounced photothermal effect was observed under NIR excitation of LSPR of the printed GNS patterns even at low laser intensities. It was found that beside the direct role of the laser intensity, this effect depends appreciably on the printing parameters, such as drop density (δ, drops/mm^2^) and number of printed layers, and, critically, on the permeability of the coated paper substrates. These results will promote the development of GNS-based printed platforms for local photothermal therapy.

## Introduction

Due to advantages over other patterning techniques, inkjet printing technology has met important challenges to pattern a broad range of functional materials with promising biomedical application [1–7]. Inks based on metal nanoparticles are widely used in inkjet printing because of their conductive and thermal properties [1,8]. Within the existing types of metal nanoparticles, the beneficial properties of gold, such as good biocompatibility, excellent resistance to oxidation and acids, and high affinity to thiol containing biomolecules promote the applicability of gold nanoparticles in nanomedicine [8–12], and in the fabrication of a broad spectrum of printed diagnostic and analytical tools [13–15]. Gold nanoparticles have also large extinction cross-sections in the range of 700–1100 nm (known as the bio-transparent window) because of localized surface plasmon resonance (LSPR). Highly localized and controlled hyperthermal effects can be obtained under excitation in resonance with the LSPR and applied for local hyperthermic treatments in life sciences [16–17].

In this study, we used well-characterized pentatwinned branched GNS with an intense LSPR extinction band in the NIR range (750–1100 nm), finely regulated through the synthesis conditions [18]. Previously, it was demonstrated that this type of GNS is photothermally active under NIR irradiation both in aqueous solutions and as monolayers grafted on dry glass surface, displaying higher temperature increase in the latter case [17,19].

Currently, great attention is paid to finding new modalities for paper as the printing substrate surpassing the limits of the conventional graphic applications. This is not unexpected considering the advantages of paper as a highly versatile and commonly employed material that combines low-cost and excellent environmental compatibility [20]. Furthermore, the physical and chemical properties of this superb printing substrate (e.g., topography, roughness, stiffness, surface energy, polarity, porosity and pore geometry) can be easily tailored by means of diverse coating materials and techniques as well as by surface treatments according to the specific needs of the envisioned applications [21–24]. In particular, metal nanoparticle films printed on paper substrates have been used in various applications that exploit electric and optical output [25–26]. The photothermal properties of printed gold nanoparticle patterns on paper substrates have not been fully explored yet, and this is the main aim of this study.

We reported recently that GNS patterns inkjet-printed onto semi-permeable paper display a substantial photothermal effect with linear dependence as a function of laser irradiance up to *I* ≈ 0.2 W/cm^2^ [27–28]. This effect was much higher than in case of previously studied GNS aqueous solutions and of monolayers of GNS on a glass surface under NIR irradiation. However, the dependence of the photothermal effect was not linear with the print density of GNS and the number of printed layers. We therefore reasoned that the physico-chemical nature of the coating of the paper substrates, e.g., paper permeability and the subsequent absorption of ink into to paper matrix may critically influence the obtained photothermal effect, and this factor is studied in this letter. Therefore, GNS with well-controlled structure and with LSPR centred at about 760 nm were synthesized, decorated with poly(ethylene glycol) thiol (PEG-SH) [18], and inkjet-printed onto three differently coated paper substrates at various drop density values (δ, drops/mm^2^) and number of layers. The dependence of the photothermal response of the printed GNS patterns on the laser intensities and printed amounts of ink is also reported.

## Results and Discussion

The stable inks were formulated by adding 1,2-ethanediol (20 vol %) and 2-propanol (10 vol %) to the aqueous PEGylated GNS solution (70 vol %) in order to adjust suitable viscosity and surface tension for inkjet printing (1.92 cP and 40 mN/m, respectively) as reported previously [27–28]. Notably, GNS decorated with PEG-SH become soluble in a variety of solvents, from water to hydrophobic ones, due to the amphiphilic nature of the poly(ethylene glycol) chains [18]. PEG-SH decoration causes LSPR of GNS to red-shift by about 10 nm, because of the slight local increase in the refractive index related to thiols grafting on the gold surface [18]. GNS decorated with PEG-SH are stable even after repeated ultracentrifugation cycles and show long time stability in the water/2-propanol/1,2-ethanediol medium used to prepare inks. The final GNS concentration (*C*_GNS_) in the prepared inks was 0.42 mg/mL. In the following, we will consider GNS patterns printed on three coated paper substrates with increasing values of porosity and permeability as measured in Gurley seconds: substrate 1 (>40000 Gurley seconds, semi-permeable, two coating layers ), substrate 2 (7360 Gurley seconds, permeable, calcium carbonate (major component) and kaolin coating) and substrate 3 (non-permeable, latex coating). The PEG-decorated GNS were inkjet-printed with varying drop density and number of layers onto the substrates. Detailed information about the synthesis, characterization, and PEGylation of the GNS, the preparation of the paper substrates and the characterization of their porosity, and the inkjet printing process is provided in Supporting Information File 1.

**Figure 1:**
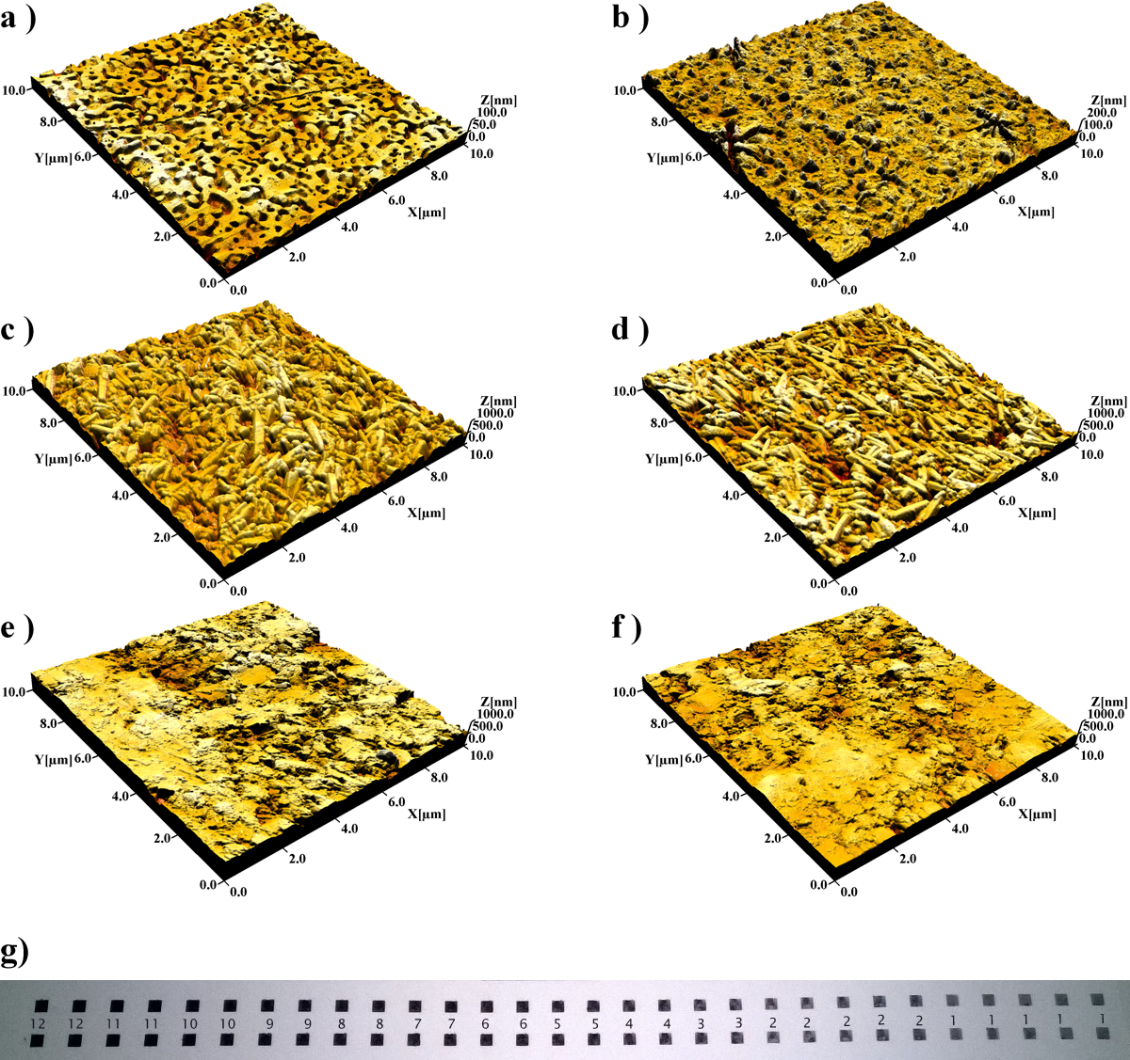
AFM topographical images (10 µm ×10 µm) of paper substrates with (left side) and without (right side) printed GNS patterns (10201 drops/mm^2^, 1 layer). The substrates are as follows: (a,b) non-permeable paper substrate; (c,d) semi-permeable paper substrate with barrier layer and (e,f) permeable paper substrate with no barrier layer. A photograph of the printed GNS patterns (4578 drops/mm^2^, 1–12 layers) on the non-permeable paper substrate is shown in panel g.

The printed GNS patterns were first characterized by means of AFM topography. Figure 1a shows an AFM topographical image of the non-permeable paper substrate with the distinctly nanostructured surface. The morphology of the surface is clearly changed after the printing of the GNS pattern showing an over-layer film with nearly complete coverage (Figure 1b). The formation of the over-layer film also resulted in a slight increase in the roughness (see Supporting Information File 2, Table S1). This indicates that GNS particles reside on the surface of the paper. Further proof of the existence of a superficial layer of GNS is given by optical micrographs (see Supporting Information File 2, Figure S1), from which is evident the reflectivity of the printed surface with respect to the non-printed one.

Figure 1c reports the AFM images of the surface of the paper coated with a semi-permeable layer of PCC, which are clearly visible as randomly oriented rod-like objects on the surface. After printing the GNS patterns no evident change can be visually gained from the morphological images (Figure 1d**)** or from the values of the roughness (see Supporting Information File 2, Table S1). In addition, the printed GNS pattern was not reflective but appeared as matt dark [27]. This indicates that the printed GNS particles did not form an uniform surface layer on the surface but rather partially penetrated inside the relatively thin (~10 µm) and porous PCC top-coating [29]. The permeable paper substrate shown in Figure 1e lacks the barrier layer that would stop the penetration of the GNS and thus it can be assumed that the GNS particles have penetrated much deeper into the paper matrix than in the case of semi-permeable paper. As a matter of fact no morphological or roughness changes were observed after the printing of the GNS patterns (Figure 1f). In addition, the printed GNS patterns were not reflective but appeared as a matt dark surface as it was the case also with the semi-permeable substrate.

**Figure 2:**
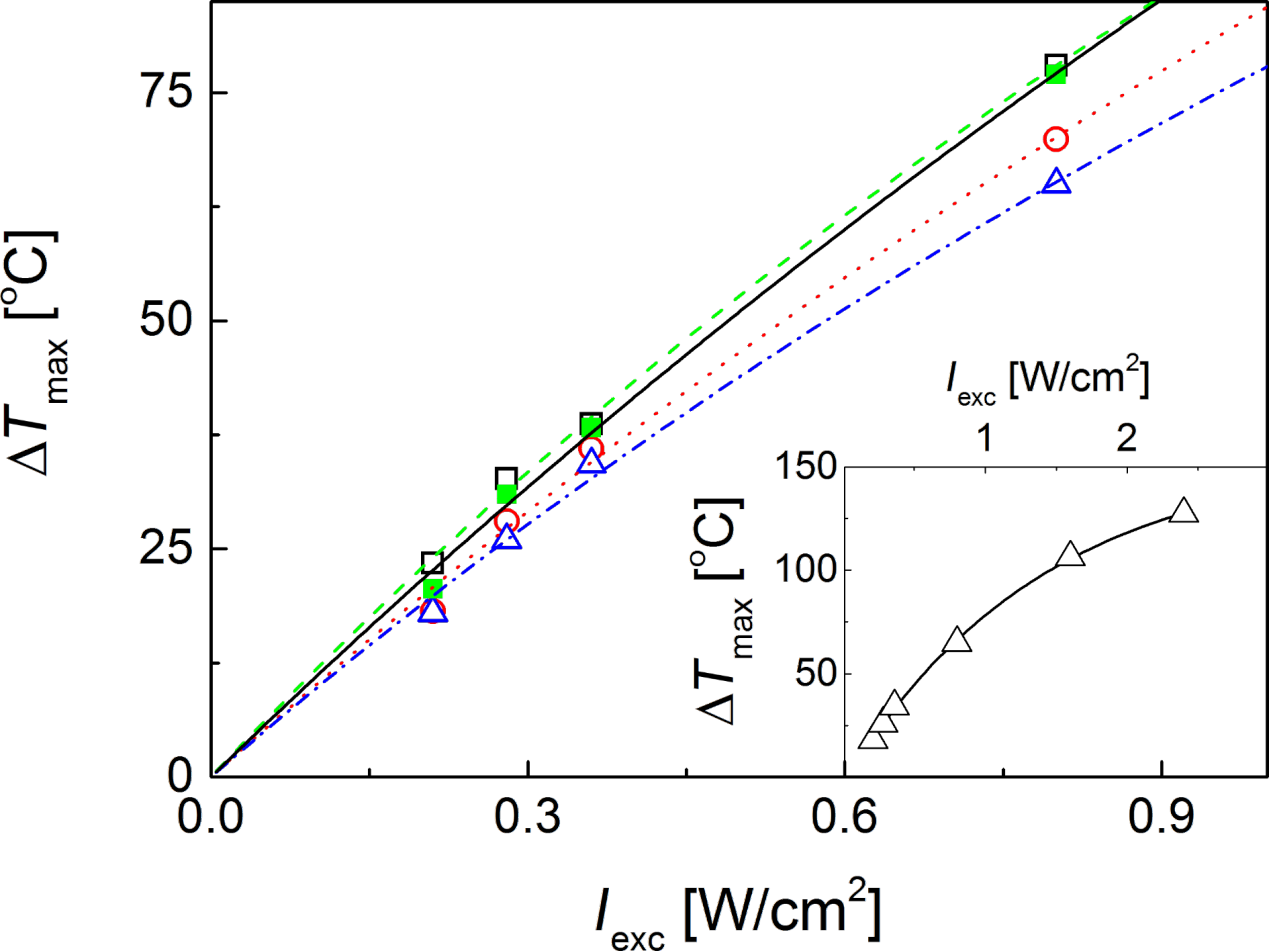
Dependence of temperature increase of GNS patterns printed on the non-permeable (filled squares), the semi-permeable (open squares and circles) and permeable (triangles) paper substrates as a function of the NIR laser intensity. The drop density of the ink for the samples was 31212 drops/mm^2^ (filled squares); 71407 drops/mm^2^ (open circles); 121203 drops/mm^2^ (open squares) and 202005 drops/mm^2^ (open traingles). Inset: dependence of temperature increase of GNS patterns printed on the permeable substrate (laser intensity above 0.8 W/cm^2^). The trial fitting function and the corresponding best fit parameters are provided in Supporting Information File 2.

The photothermal effect of printed GNS patterns under ambient conditions was then induced by irradiating them at λ = 800 nm, close to the NIR LSPR (the absorption maximum is 770 nm, see Supporting Information File 1, Figure S1) of the PEGylated GNS as measured in solution. After an initial steep temperature increase, a plateau was reached in all cases after approximately 15 s (see Supporting Information File 2, Figure S2 and Figure S3). The increase of temperature (Δ*T*) was measured as a function of the laser intensity. The results shown in Figure 2 indicate that a significant photothermal effect can be reached even at relatively low laser intensities for all paper substrates and for a wide range of drop density. After an initial linear temperature increase, a saturation effect sets in at about *I*_exc_ = 0.5 W/cm^2^. This behaviour can be rationalized in terms of a model that accounts for the localized light absorption by the printed pattern, its energy conversion to heat and subsequent dissipation of the heat to the surrounding environment (see Supporting Information File 2). As a comparison, control experiments performed by irradiating the blank substrate under the same conditions showed a negligible photothermal effect (Δ*T* ≤ 0.4 °C).

The much higher temperature increase achieved on the printed GNS patterns under ambient conditions compared to the values previously reported for GNS in aqueous solutions [17,30] is likely due to the absence of the thermal dissipation from the bulk solvent (water) that has a high thermal capacity compared to air (see the model provided in Supporting Information File 2).

In comparison with the semi-permeable and the non-permeable substrates, a lower NIR-induced photothermal effect was observed on GNS patterns printed on permeable substrate (triangles), even if a higher amount of ink was printed (Figure 2). This can be explained by the lower efficiency of this permeable substrate in preventing the particles from penetrating deep into the paper matrix and therefore, higher nanoparticles concentrations are needed to obtain the same temperature increase as found for the semi-permeable and non-permeable substrates (see Figure 2, circles). It is noteworthy that the GNSs printed on the non-permeable substrate showed a hyperthermic effect very similar to that on the semi-permeable substrate over the whole range of irradiation wavelengths, even if the drop density was one fourth (see Figure 2, open and filled squares).

**Figure 3:**
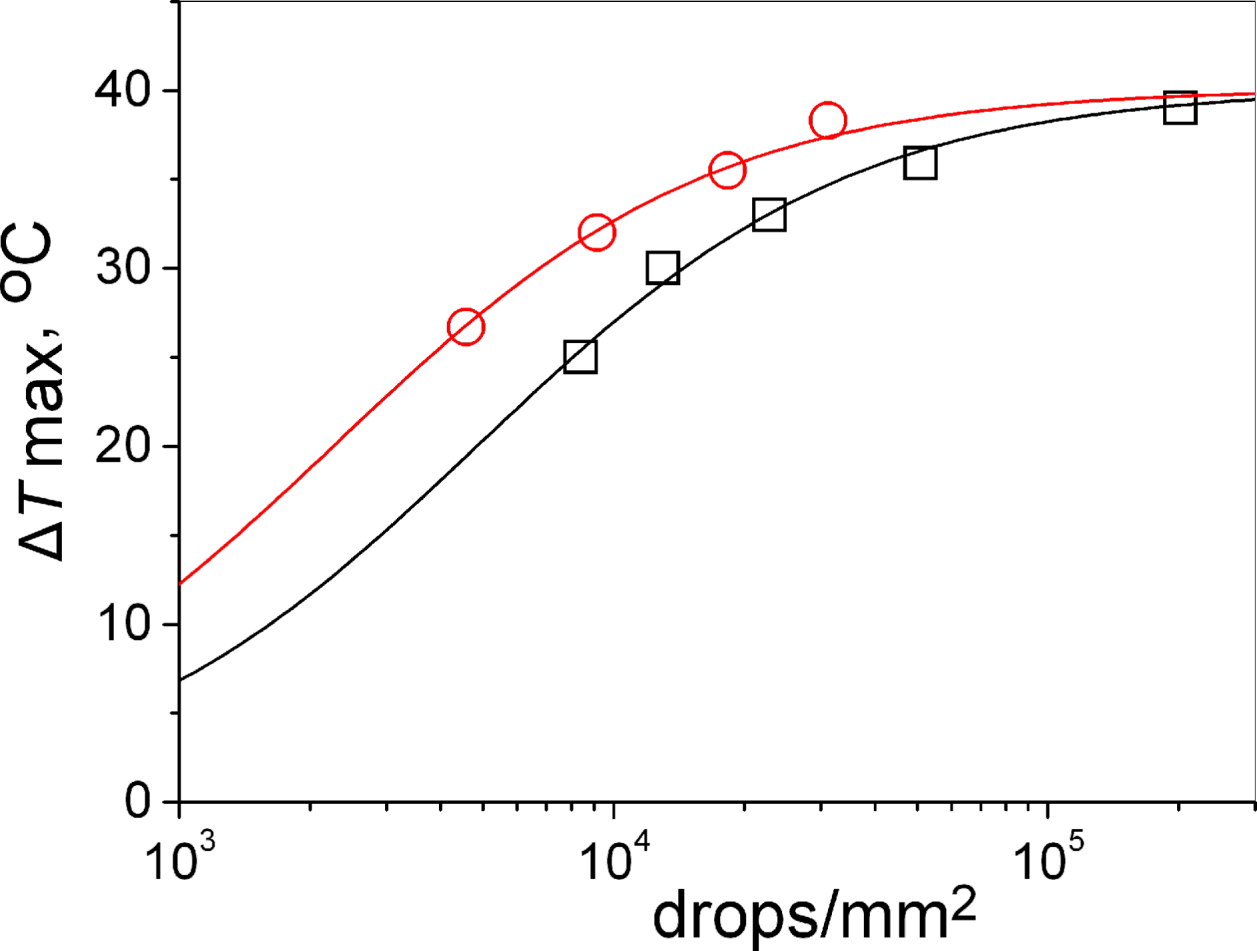
Photothermal effect as a function of the amount of printed GNS for the non-permeable substrate (open circles) and the permeable substrate (open squares). Laser intensity was 0.36 W/cm^2^. The solid lines are the best fit of the trial function (see Supporting Information File 2) to the data.

This behaviour is likely because of the negligible penetration of nanoparticles into the paper structure. In order to better elucidate this effect, we have studied the role of the gold density on the hyperthermic efficiency of the patterns printed on different types of paper substrates. The temperature increase measured as a function of the drop density is shown in Figure 3 (for thermal load kinetics on the non-permeable substrate please refer to Supporting Information File 2). The thermal response increases also with the drop density with a marked non-linear response for drop density values above 5000–10000 drops/mm^2^.

It is notable that the maximum value of the temperature increase at very high drop densities is very similar, irrespective of the nature of the paper substrate. This indicates that at a high drop density, the light is completely absorbed by a thin layer of printed GNS. Intensity losses can be caused by an inner filtering effect of the GNS and by scattering of the paper substrate. Therefore, in the previously developed simple model [27] it was shown that the intensity loss is predominantly caused by inner filtering, while the contribution of scattering of paper substrate is not substantial in the case of gold nanoparticles where absorption is dominant (see also Supporting Information File 2).

**Figure 4:**
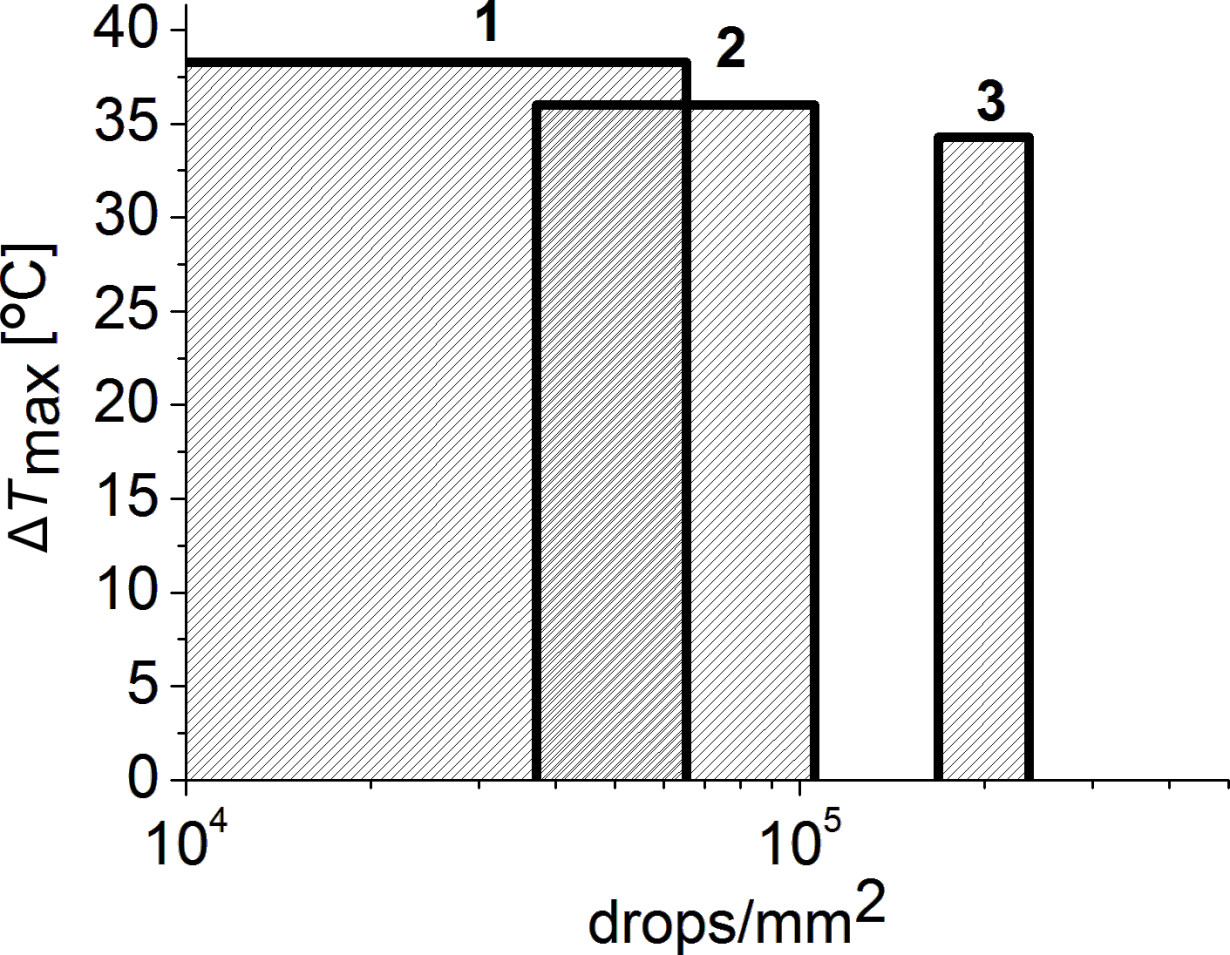
Comparison of the different ink amounts needed to reach similar photothermal effect (Δ*T* ≈ 35 °C) of on GNS patterns printed onto three differently coated paper substrates: non-permeable (1); semi-permeable (2), and permeable (3). The laser intensity was 0.36 W/cm^2^.

However, at lower drop density values different amounts of printed GNS are needed on coated paper substrates with different porosity in order to reach similar temperatures under the same laser intensity. As shown in Figure 4, we can reach Δ*T* ≈ 35 °C with about a sixth of gold on the least permeable substrate compared to the most permeable one.

## Conclusion

In the present study, inks based on stable PEGylated GNS were fabricated and successfully inkjet-printed onto three types of paper substrates with different coatings and permeability. All these printed patterns showed a pronounced local photothermal effect (Δ*T* > 30 °C) under NIR irradiation at low laser intensities (*I*_exc_ < 0.8 W/cm^2^). Apart from the printing parameters (drop density and number of layers), the porosity of the coated paper substrates has a significant impact on the photothermal properties of the printed patterns. On the non-permeable substrate, a significant photothermal effect was achieved with a sixth of ink compared to the permeable substrate. This finding is particularly useful in definining the minimum amount of gold to be printed on smart gold-based devices. Moreover, it should be noted, that the non-permeable paper displays also good biocompatibility at least for in-vitro applications, due to its latex coating [24]. Therefore, further steps of this research will employ non-permeable paper as printing substrate, and our future study will be focused on studying antibacterial and antifungal properties of these surfaces, and on the NIR-triggered release of drugs bound to printed gold surface as an additional and valuable effect.

## Supporting Information

File 1 Full experimental data.

File 2 Additional AFM and NIR irradiation data.
