# Supplementary material for: Photothermal effect of gold nanostar patterns inkjet-printed on coated paper substrates with different permeability
Source: Beilstein J Nanotechnol. 2016 Oct 19;7:1480–5. doi: 10.3762/bjnano.7.140 (PMC5082577; doi:10.3762/bjnano.7.140)
Supplement: File 1 — Full experimental data. [file Beilstein_J_Nanotechnol-07-1480-s001.pdf]

Supporting Information File

for

Photothermal effect of gold nanostar patterns inkjet-printed on coated paper substrates with different permeability

Mykola Borzenkov*[1], Anni Määttänen*[2], Petri Ihalainen[2], Maddalena Collini[1], Elisa Cabrini[3], Giacomo Dacarro[3], Piersandro Pallavicini[3] and Giuseppe Chirico[1]

Address: [1]Department of Physics "G. Occhialini", Nanomedicine Center, University of Milano-Bicocca, piazza dell'Ateneo Nuovo, 20126, Milan, Italy; [2]Laboratory of Physical Chemistry, Center of Functional Materials, Åbo Academi University, Porthaninkatu 3-5, 20500, Turku, Finland and [3]Department of Chemistry, University of Pavia, viale Taramelli 12, 27100, Pavia, Italy

Email: Mykola Borzenkov* - mykola.borzenkov@unimib.it;
Anni Määttänen* - annimaa@gmail.com
* Corresponding author

Full experimental data

1. Chemicals

Poly(ethylene glycol) thiol (average molecular weight <M_W> = 5000 g/mol; PEG_{5000}-SH), *N*-dodecyl-*N*,*N*-dimethyl-3-ammonio-1-propanesulfonate (laurylsulphobetaine, LSB), chloroauric acid, ascorbic acid, silver nitrate, sodium borohydride, 2-propanol, 1,2-ethanediol, ethanol were purchased from Sigma-Aldrich (NL) and used as received.

2. GNS synthesis

GNS solutions were obtained in 10.0 mL portions by seed growth synthesis with the zwitterionic surfactant LSB, as we have already described [Pallavicini 2011]. The reactants concentrations were chosen so to obtain the LSPR of the prevalent product (pentatwinned branched GNS) in the 750-760 nm range, as described [Pallavicini 2011; Casu 2012]. After the growth process, GNS solutions were ultracentrifuged, the supernatant solvent discarded, and the GNS cake completely redissolved in 10.0 mL of bidistilled water. These are the solutions used for coating with PEG.
Briefly: Seed solution (a) was prepared in a 20 mL vial by adding LSB solution (5.0 mL) in water (0.20 M) and $HAuCl_4$ 5×10^{-4} M (5.0 mL) in water. Then an ice-cooled solution of $NaBH_4$ (600 μL, 0.01M) in water was added. The vial was gently hand-shaken for 10 s, and the solution assumed the brown-orange color of Au nanoseeds. Growth solution (b): This was prepared in a 20 mL vial by using LSB solution (5.0 mL) in water at the same concentration chosen for the seed preparation (0.2M) and $AgNO_3$ (180 μL, 0.004m) in water. To this solution, aqueous $HAuCl_4$ (5.0 mL, 0.001M) was added.
I 90 μL volume of 0.0788M aqueous ascorbic acid solution was then added.
II 12 μL volume of solution (a) was added to solution (b). Growth was complete in 1-2 hours.

References

Casu, A et al. *Chemistry Eur. J.* **2012**, *18*, 9381-9390.
Pallavicini, P et al. *Chem. Commun.* **2011**, *47*, 1315-1317.

3. Coating of GNS with PEG-SH

This was carried out by simultaneously adding of 200 μL of a 10^{-3} M of aqueous solution of PEG-SH (average molecular weight <M_W> = 5000 g/mol) to 100 mL of a GNS solution prepared as described. The obtained solution was allowed to equilibrate for 3 hours at room temperature while gently shaken on a reciprocating shaker. Excess of PEG-SH was removed by ultracentrifugation (25 min, 13000 rpm), supernatant discarding, pellet redissolution in 10mL of bi-distilled water. The cleaning cycle was repeated two more times to assure complete elimination of unbound PEG-SH and of any LSB. The latter is completely removed also from the GNS surface after such treatment. More concentrated GNS solutions were prepared using larger GNS volumes in the PEGylation step (100 mL instead of 10 mL), and redissolving the GNS pellet after the last ultracentrifugation cycle in 10 mL of bi-distilled water.

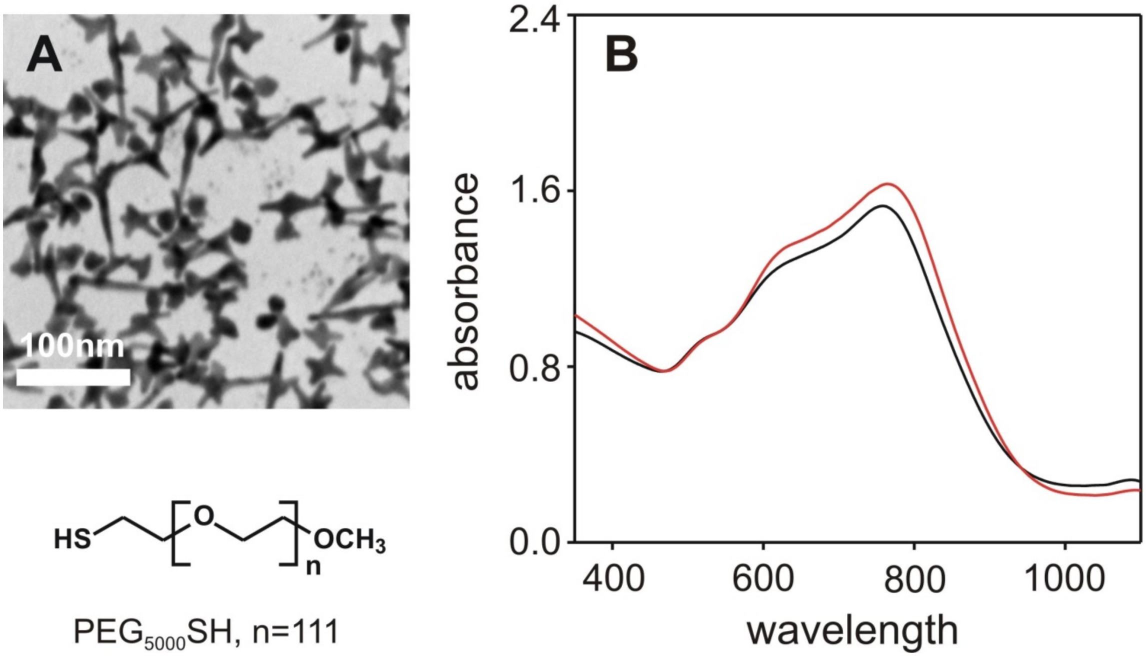

Figure S1: Structural and spectroscopic characterization of GNS. Panel A: exemplary TEM image of PEGylated GNS. Panel B: extinction spectra of bare (black line) and PEGylated GNS (red line) showing the red shift of LSPR.

References

Borzenkov, M. et al. *Proc. SPIE* **2016**, *9887*, 98872C.
Borzenkov, M. et al. *ACS Appl. Mater. Interfaces* **2016**, *8*, 9909-9916.

4. Printing substrates

Paper substrate 1: Semi-permeable coating

The proprietary multilayer laboratory-coated paper substrate was used as a semi-permeable paper substrate. It consists of the following layers: precoated base paper, blade-coated Kaolin layer, barrier coating (blend of styrene acrylate (SA) latex and precipitated calcium carbonate pigment (PCC) pigments), and a top coating (blend of PCC pigments and styrene–butadiene binder) [1]. This paper substrate has been specially designed for printed electronics and contains a barrier layer underneath thin porous top coating layer [2]. The top coating enhances the print resolution and adhesion as well as reduces e.g. ink smearing. The barrier layer prevents the functional inks from penetrating too deep inside the paper structure (~average pore diameter 90 nm, top coating thickness < 10 µm), and the ink penetration is limited to the top coating matrix. This substrate was fabricated utilizing commonly used coating components in the papermaking industry. Therefore, the substrate is recyclable like any other ordinary coated paper used in graphical printing industry. More detailed information about the substrate, its fabrication and physicochemical properties is given elsewhere [3].

Paper substrate 2: Permeable coating

Commercial sheet-fed offset (SFO) paper coated with calcium carbonate (major component) and Kaolin was used as a permeable paper substrate. The paper substrate does not contain any barrier layer, thus allowing the penetration of the ink deep into the paper matrix [4].

Paper substrate 3: Non-permeable coating

Non-permeable paper substrate was fabricated by a rod-coating of a multi-layer curtain-coated barrier base paper with a latex layer. The base paper substrate has excellent barrier properties against water and solvent penetration [5]. Latex-coating contained an aqueous dispersion of a latex blend consisting of carboxylated styrene butadiene acrylonitrile (ABS) copolymer and modified polystyrene (PS) copolymer with the final ABS:PS weight ratio of 3:2. In the PS: ABS latex blend, the PS component provides blocking resistance, mechanical strength and integrity to the film, while the ABS component acts as a film-forming component. The nanostructured surface texture was created by irradiating the latex-coated paper with a short-wavelength infrared heater (IRT systems, Hedson Technologies AB, Sweden) for 60 s [6].

The air permeance was measured using L&W Air Permeance Tester according to the Bendtsen method to evaluate the porous nature of the substrates. The air permance is given in Gurley seconds. Gurley numbers tend to increase with decreasing air-permeability of the paper, since what one is measuring is the time required for a selected volume of air to leak through a defined area (100 cm^2). In addition, the air permeance for highly permeable uncoated office copy is shown as comparison.

Substrate 1: > 40000 Gurley seconds
Substrate 2: 7360 Gurley seconds
Substrate 3: non-porous
Uncoated office copy paper: 17.5 Curley seconds

References

[1] Määttänen, A., Ihalainen, P. et al. *Colloid and Surfaces A: Physicochemical and Engineering Aspects* **2010**, *367*, 76-84.
[2] Bollström, R. et al. *Org. Electronics* **2010**, *10*, 1020-1023.
[3] Borzenkov, M. et al. *Proc. SPIE* **2016**, *9887*, 98872C.
[4] Ihalainen, P. et al. *Industrial and Engineering Chemistry Research* **2012**, *51*, 6025-6036.
[5] Sarfraz, J. et al. Sensors & Actuators B: Chemical 2012, *173*, 868-873.
[6] Juvonen, H. Et al. *Colloids and Surfaces B: Biointerfaces* **2015**, *136*, 527-535.

5. Inkjet printing of GNS ink

Inkjet printing of GNS ink was performed with Dimatix Materials Printer (DMP-2800, FUJIFILM Dimatix, Inc, Santa Clara, USA). The printing was performed in ambient conditions using a single nozzle with a nominal droplet volume of 10 pL, 16 ± 3 V firing voltage, 2 kHz firing frequency and a custom waveform to ensure optimal droplet formation. Various drop spacing (ds, i.e. center to center distance drops of 5 μm - 30 μm) and layer counts (1–7) were used to print the GNS patterns over surfaces with different sizes (A: 1 mm^2, 4 mm^2, 9 mm^2 and 16 mm^2).

Table S1: The compliance of drop spacing parameters with amount of printed ink.

Drop Spacing, DS	Drops/mm^2	Volume, µL/mm^2
5	40401	0.40401
7.1*	20284	0.202839
10	10201	0.10201
15	4578,78	0.04578
20	2601	0.02601
21.2*	2317,5	0.023175
25	1681	0.01681
30	1178,78	0.0117878

References

Borzenkov, M. et al. *Proc. SPIE* **2016**, *9887*, 98872C.
Borzenkov, M. et al. *ACS Appl. Mater. Interfaces* **2016**, *8*, 9909-9916.

6. Atomic force microscopy

An NTEGRA PRIMA (NT-MDT, Russia) atomic force microscope (AFM) was used for topographical analysis and thickness determination of the inkjet-printed GNS films. The images (1024 pixels × 1024 pixels) were captured in intermittent-contact mode in ambient conditions using silicon cantilevers with a nominal tip radius of 10 nm (Model: NSG 10, NT-MDT, Russia). The scanning rate and damping ratio were 0.2–0.3 Hz and 0.6–0.7, respectively. Image analysis was done using SPIP^{TM} image analysis software (Image Metrology, Denmark).

7. Near infrared irradiation of printed GNS patterns

The patterns were irradiated at λ = 800 nm (Tsunami, Spectra Physics, CA, pulse repetition rate 80 MHz, pulse width 200 fs). The substrate temperature changes were monitored by means of a Thermovision camera (FLIR, E40, USA) with supporting software. The temperature was monitored in time on a Region of Interest (ROI) comprising the irradiated area and the maximum temperature within the ROI was taken as the measure of the temperature increase on the pattern induced by the NIR irradiation until the steady-state was reached. All the temperature increase data refer to the difference between this steady-state value, measured under continuous irradiation, and the room temperature.
