# Supplementary material for: Photothermal effect of gold nanostar patterns inkjet-printed on coated paper substrates with different permeability
Source: Beilstein J Nanotechnol. 2016 Oct 19;7:1480–5. doi: 10.3762/bjnano.7.140 (PMC5082577; doi:10.3762/bjnano.7.140)
Supplement: File 2 — Additional AFM and NIR irradiation data. [file Beilstein_J_Nanotechnol-07-1480-s002.pdf]

Supporting Information File

for

Photothermal effect of gold nanostar patterns inkjet-printed on coated paper substrates with different permeability

Mykola Borzenkov*[1], Anni Määttänen*[2], Petri Ihalainen[2], Maddalena Collini[1], Elisa Cabrini[3], Giacomo Dacarro[3], Piersandro Pallavicini[3] and Giuseppe Chirico[1]

Address: [1]Department of Physics "G. Occhialini", Nanomedicine Center, University of Milano-Bicocca, piazza dell'Ateneo Nuovo, 20126, Milan, Italy; [2]Laboratory of Physical Chemistry, Center of Functional Materials, Åbo Academi University, Porthaninkatu 3-5, 20500, Turku, Finland and [3]Department of Chemistry, University of Pavia, viale Taramelli 12, 27100, Pavia, Italy

Email: Mykola Borzenkov* - mykola.borzenkov@unimib.it;
Anni Määttänen* - annimaa@gmail.com
* Corresponding author

Additional AFM and NIR irradiation data

Table S1: Values for selected roughness parameters for each sample.[a]

Sample	Sq (nm)	Scl (nm)	Sz (nm)	Sdr (%)
**Semi-permeable**	**90 ± 10**	**213 ± 25**	**779 ± 53**	**76 ± 5**
**Semi-permeable with GNS**	**81 ± 5**	**201 ± 23**	**835 ± 35**	**72 ± 5**
**permeable**	**41 ± 7**	**268 ± 27**	**406 ± 63**	**24 ± 5**
**Permeable with GNS**	**43 ± 4**	**259 ± 40**	**408 ± 27**	**24 ± 7**
**Non-permeable**	**19 ± 4**	**120 ± 45**	**133 ± 15**	**8 ± 2**
**Non-permeable + GNS**	**24 ± 2**	**134 ± 17**	**220 ± 25**	**26 ± 5**

Sq = RMS rougness, Scl = correlation length, i.e., average lateral spacing of height features, Sz = maximal height difference, Sdr = relative increase in surface area compared to flat (Sdr = 0%) surface**.**

IR sintered GNS patterns on non-permeable paper substrate

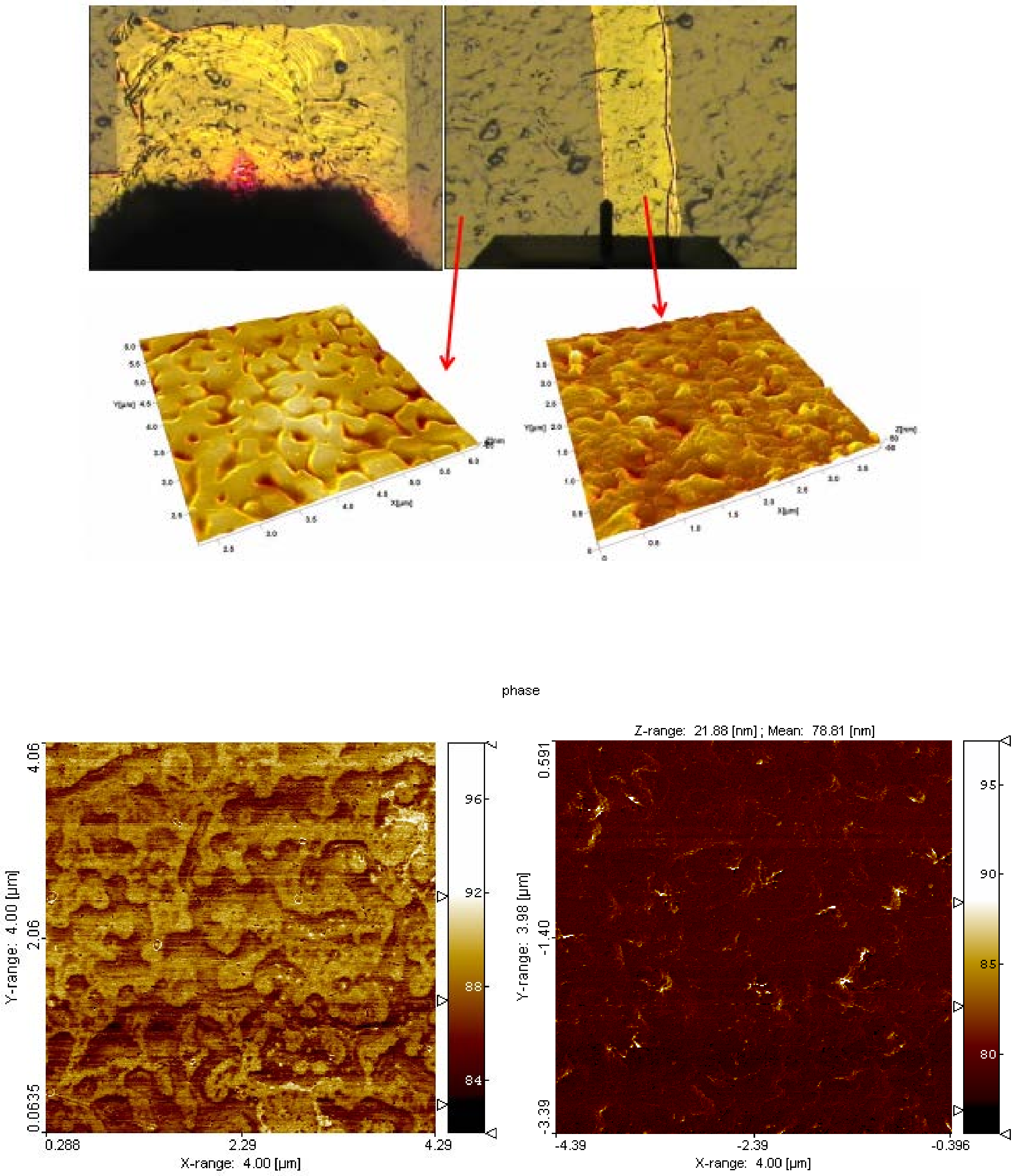

Two phases one phase (Full coverage)

Figure S1: Optical micrographs of IR-sintered GNS patterns on non-permeable paper substrate.

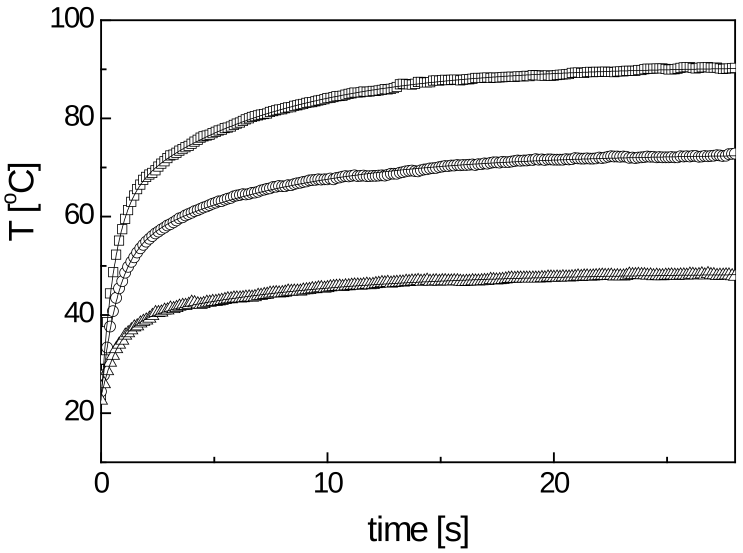

Figure S2: The NIR-induced temperature increase of GNS patterns printed on semi-permeable substrate (71407 drops/mm^2). The solid lines are the double exponential best fit functions to the data, allowing to extrapolate the maximum temperature increase, ΔT. The data correspond to different irradiation intensity values: I = 0.8 W/cm^2 (square), I = 0.54 W/cm^2 (circles), I = 0.3 W/cm^2 (triangles). Irradiation starts immediately at time t = 0.

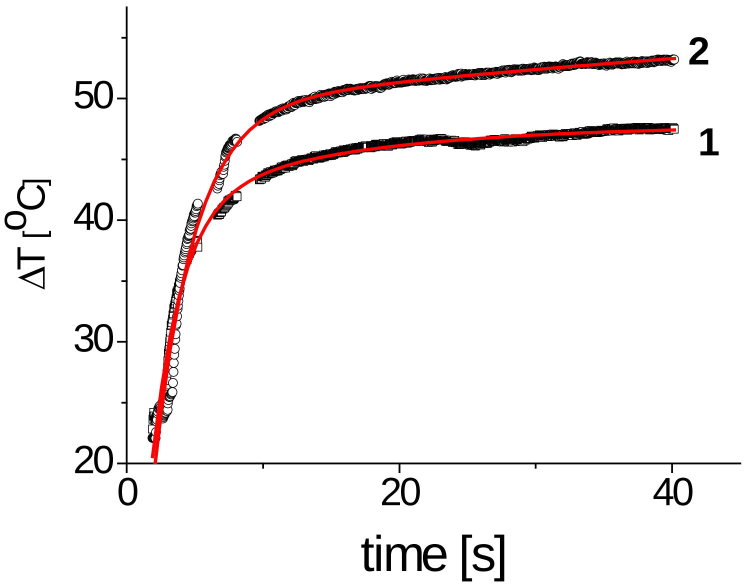

Figure S3: The NIR induced temperature increase of printed on non-permeable substrate GNS patterns. The solid red lines are the double exponential best fit functions to the data. Printed amount of GNS inks was 4578 drops/mm^2 (1) and 9156 drops/mm^2 (2). Laser intensity: 0.36 W/cm^2.

Mathematical model of the thermal load kinetics (fitting parameters of Figures 2–3)

The dependence of the maximum temperature difference induced by NIR irradiation is well described by a linear trend up to low laser intensities, according to Equation 1:

$$\Delta T_{max} \sim \Delta I \simeq I_{exc}\left(1-e^{C\varepsilon\Delta x}\right) = \beta I_{exc} \qquad (1)$$

where ΔI is the loss in excitation intensity due to nanoparticles absorption, written here as the difference between the excitation and the transmitted beam, C is the GNS concentration in inks, Δx is the layer thickness and ε is related to the absorption cross section of GNS. Equation 1, however, would predict a linear behavior as a function of I_{exc} for all the intensity range. A second mechanism that can drain heat from the irradiated GNS layer is due to the exchange with the surrounding environment. The experimental maximum temperature increase would then be given by an implicit Equation 2 that, at the first order, can be stated as:

$$\Delta T_{max}^{exp} \simeq \Delta T_{max} - \kappa_{eff}(T)\Delta T_{max}^{exp} \qquad (2)$$

where $\kappa_{eff}(T)$ is an effective adimensional parameter, related to the thermal conductivity of the environment divided by the mass and the specific heat of the GNS, for unit time and area. Its temperature dependence, primarily due to the air thermal conductivity, can be obtained by a series expansion as a function of the temperature increase, $\kappa_{eff}(T) = \kappa_{eff}^0 + \frac{\partial \kappa_{eff}(T)}{\partial T}\Delta T + o(\Delta T^2)$, that inserted in Equation 2 leads to Equation 3:

$$\Delta T_{max}^{exp} \simeq \frac{\Delta T_{max}}{1+\kappa_{eff}^0 + \frac{\partial \kappa_{eff}}{\partial T}\Delta T_{max}} = \frac{G\beta I_{exc}}{1+\kappa_{eff}^0 + \frac{\partial \kappa_{eff}}{\partial T}G\beta I_{exc}} = \frac{K I_{exc}}{1+I_{exc}/I_{sat}} \qquad (3)$$

where G takes into account the correct proportionality constant in Equation 1 and has the unit of K·m^2/W.

By combining Equation 1 and Equation 3, we can describe also the dependence of ΔT_{max}^{exp} upon the concentration C:

$$\Delta T_{max}^{exp} = \frac{I_{exc}G\left(1-e^{-C\varepsilon\Delta x}\right)}{1+\kappa_{eff}^0 + \frac{\partial \kappa_{eff}}{\partial T}I_{exc}G\left(1-e^{-C\varepsilon\Delta x}\right)} = \frac{\left(1-e^{-C\varepsilon\Delta x}\right)}{\frac{1+\kappa_{eff}^0}{G I_{exc}} + \frac{\partial \kappa_{eff}}{\partial T}\left(1-e^{-C\varepsilon\Delta x}\right)} \qquad (4a)$$

The maximum temperature increase for different substrates is fitted to Equation 4a as a function of the concentration by means of the trial function:

$$\Delta T_{max}^{exp} = \frac{1-e^{-AC}}{B+D\left(1-e^{-AC}\right)} \qquad (4b)$$

Continuous lines in Figure 2 in the main text are obtained by fitting with Equation 3 with the following parameters:

Paper substrate	K (K·cm^2/W)	I_{sat} (W/cm^2)
Latex coating (31212 drops/mm^2)	113 ± 4	4.7 ± 0.9
Semi-permeable (121203 drops/mm^2)	122 ± 4	3.2 ± 0.7
Semi-permeable (71407 drops/mm^2)	104 ± 8	4.3 ± 2.5
Permeable	100 ± 7	3.5 ± 1.5

The large uncertainties are due to the low laser powers used for the irradiation, therefore giving a poor estimate of the saturation intensity, whereas the *K* parameter, which is related to the steep, is better estimated.

Continuous lines in Figure 3 are obtained by global fitting the data to Equation 4b with B = $2.8\cdot10^{-5}$ K^{-1}, D = 0.025 K^{-1} as shared parameters and whereas A is determined for each substrate. A is of the order of 10^{-7} mm^2, and though determined with a large uncertainty, its value for the permeable substrate is half of that for the semi-permeable one.
